# Treating ROP: how and when

**Published:** 2017

**Authors:** Graham Quinn, Clare Gilbert

**Affiliations:** Professor Emeritus of Ophthalmology: The Children's Hospital of Philadelphia, Wood Center, Philadelphia, USA.; Professor of International Eye Health and Co-director: International Centre for Eye Health, London School of Hygiene & Tropical Medicine, London, UK.

**Figure:**
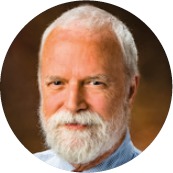
Graham Quinn

**Figure:**
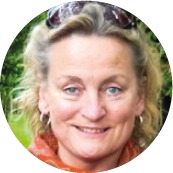
Clare Gilbert

**Laser treatment of ROP is highly effective. However, special care should be taken when treating preterm or newborn infants, and long-term follow-up is essential. There are also new treatments on the horizon, particularly in cases where laser treatment is not possible or has failed.**

**Figure:**
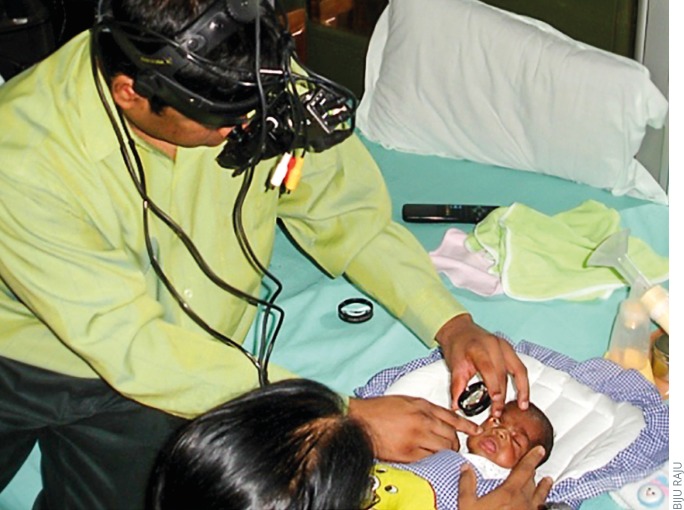
Ophthalmologist checks whether ROP treatment has been successful. INDIA

## Indications for treatment

The Early Treatment of Retinopathy (ROP) trial (ET-ROP)^1^ clearly showed that earlier laser treatment gives better results than waiting until ‘threshold disease’ develops. The ET-ROP indications for treatment use a combination of zone, stage and whether plus disease or aggressive posterior ROP is present (Figure 4, pp. 58).

ROP in zone I has the worst prognosis and so requires treatment at an earlier stage than ROP in zone II or III. The presence of plus disease also indicates a poorer prognosis. Eyes with plus disease and aggressive posterior ROP also have a poorer prognosis.

## Laser treatment

**Figure 1:**
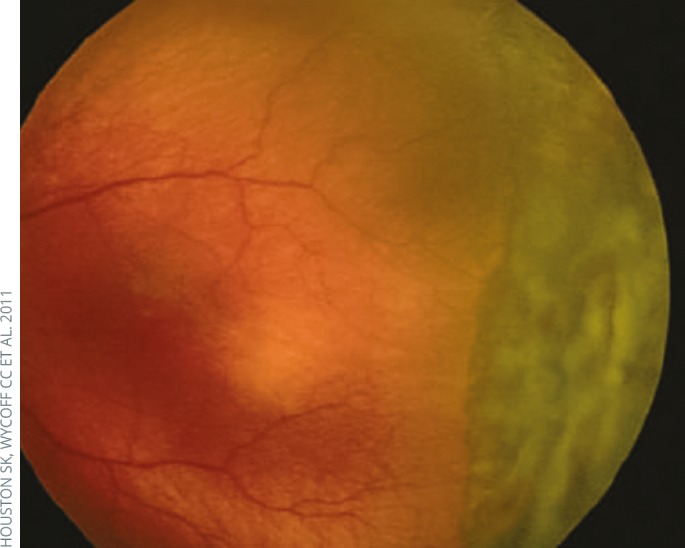
Photocoagulation pattern for ROP. The burns should be almost confluent.

The mainstay of treatment for severe ROP is peripheral retinal photocoagulation, delivered by laser. Only the avascular retinal periphery should be treated. The laser burns should be light and almost confluent (Figure 1). Laser treatment requires a trained and highly skilled ophthalmologist.

Treatment is painful and should be given under topical anaesthesia with or without sedation, or under general anaesthesia. It is essential that the infant is monitored closely during treatment. A neonatologist or trained neonatal nurse must be present.

Babies should be followed up closely after treatment (after 1 week initially) to ensure that the ROP is regressing and that treatment of the peripheral retina is complete, with no skip areas (areas of untreated retina).

Further treatment should be given if the ROP is not regressing, including to skip areas.

## Other treatment

Agents which block vascular endothelial growth factor (VEGF), which stimulates new vessel growth, are being explored as a treatment for ROP.^2^ Although these agents, which are given by intravitreal injection, can give rapid short-term resolution of ROP, there are concerns about the long-term complications in the eye and possible systemic complications. For this reason, anti-VEGF agents are only recommended when laser treatment is not possible (i.e., the baby is too sick, the pupils do not dilate, or there is intravitreal haemorrhage) or when extensive laser treatment has failed. Parents should be fully informed about the risks before treatment and must give their consent.

## Follow-up after treatment

All babies treated for ROP should have long-term follow-up visits to detect and manage the eye conditions which frequently develop in these children (pp. 62–64).

## References

[1] Good WV and the Early Treatment for Retinopathy of Prematurity Cooperative Group. Final results of the Early Treatment for Retinopathy of Prematurity (ETROP) randomized trial. Trans Am Ophthalmol Soc. 2004;102: 233–48. 15747762 PMC1280104

[2] Klufas MA Chan RV Intravitreal anti-VEGF therapy as a treatment for retinopathy of prematurity: what we know after 7 years. J Pediatr Ophthalmol Strabismus. 2015;52(2):77–84. 25798707 10.3928/01913913-20150216-01

